# Drug therapy for myocarditis induced by immune checkpoint inhibitors

**DOI:** 10.3389/fphar.2023.1161243

**Published:** 2023-05-25

**Authors:** Yihao Wu, Yizhou Xu, Linhao Xu

**Affiliations:** ^1^ Department of Cardiology, Affiliated Hangzhou First People’s Hospital, Zhejiang University School of Medicine, Hangzhou, Zhejiang, China; ^2^ Department of Laboratory Medicine, Affiliated Hangzhou First People’s Hospital, Zhejiang University School of Medicine, Hangzhou, Zhejiang, China

**Keywords:** immune checkpoint inhibitors, programmed cell death 1, myocarditis, cardiotoxicity, inflammation, fibrosis, cytotoxic T-lymphocyte antigen 4

## Abstract

Immune checkpoint inhibitors (ICIs), including cytotoxic T-lymphocyte antigen 4 (CTLA-4), programmed cell death 1 (PD-1), and its ligand 1 (PD-L1), have improved the survival in multiple types of cancers; however, ICIs may cause cardiovascular toxicity. Although rare, ICI-mediated cardiotoxicity is an extremely serious complication with a relatively high mortality. In this review, we discuss the underlying mechanism and clinical manifestations of cardiovascular toxicity induced by ICIs. According to previous studies, multiple signaling pathways are involved in myocarditis induced by ICIs. Further, we summarize the clinical trials of drugs for the treatment of ICI-associated myocarditis. Although these drugs have shown the beneficial effects of alleviating cardiac function and reducing mortality rates, their efficacy is not optimal. Finally, we discuss the therapeutic potential of some novel compounds as well as the underlying mechanisms of their action.

## 1 Introduction

Although some of the main cancer therapies, such as chemotherapy and radiation therapy, have significantly improved long-term survival rates, an increased risk of cardiac dysfunction has been observed in cancer survivors (Kostakou et al., 2019). Immune checkpoint inhibitors (ICIs), which represent the most notable breakthrough in cancer therapy, have shown significant clinical efficacy with reduced adverse events (Gong et al., 2018). Although rare, cardiovascular toxicities associated with ICIs are often serious complications with a relatively high mortality (Lyon et al., 2018).

The therapeutic mechanism of ICIs is based on targeting certain immunoregulatory signaling molecules, including cytotoxic T-lymphocyte antigen 4 (CTLA-4), programmed cell death 1 (PD-1), and its ligand 1 (PD-L1), which activate T cells and inhibit the growth of tumor cells (Ribas and Wolchok, 2018). So far, eight agents, including one CTLA-4-blocking antibody (ipilimumab), three PD-1-blocking antibodies (nivolumab, pembrolizumab, cemiplimab, and dostarlimab), and three PD-L1-blocking antibodies (atezolizumab, avelumab, and durvalumab) have been approved for clinical use by the United States Food and Drug Administration (US FDA) (Wu et al., 2022). However, reports on immune-mediated cardiovascular toxicities have been increasing, and up to 80% of patients treated with ICIs may experience such adverse events and even death (Mir et al., 2018). Indeed, the rate of various adverse cardiac events, such as cardiovascular arrest, cardiogenic shock, and myocardial infarction, in patients treated with ICIs is four times greater than that in patients without ICI treatment (Moslehi et al., 2018). Therefore, multiple immunosuppressant drugs, such as methylprednisolone, prednisone, infliximab, anti-thymocyte globulin, mycophenolate mofetil rituximab, and tacrolimus, that attenuate the cardiotoxicity induced by ICIs have been widely investigated (Brahmer et al., 2018; Chen et al., 2021a; Kennedy et al., 2022); however, the efficacy of these drugs is not optimal and their use requires immediate termination of ICI treatment.

We searched the PubMed, Embase, Cochrane Library, and China National Knowledge Infrastructure (CNKI) electronic databases for the following terms: 1) “immune checkpoint inhibitors” or “PD-1” or “PD-L1” or “CTLA-4” and 2) “myocarditis” or “cardiotoxicity”. The searches were limited to studies published in English or Chinese. The final literature searches were performed on 5 Dec 2022.

In this review, we summarize the pathogenic mechanism of ICI-induced cardiotoxicity and evaluate the commonly used cardioprotective drugs. We also review the putative molecular mechanism underlying the effects of new drugs used to treat cardiotoxicity induced by ICIs in preclinical studies.

## 2 Molecular mechanisms of cardiotoxicity induced by CTLA-4 axis

**FIGURE 1:**
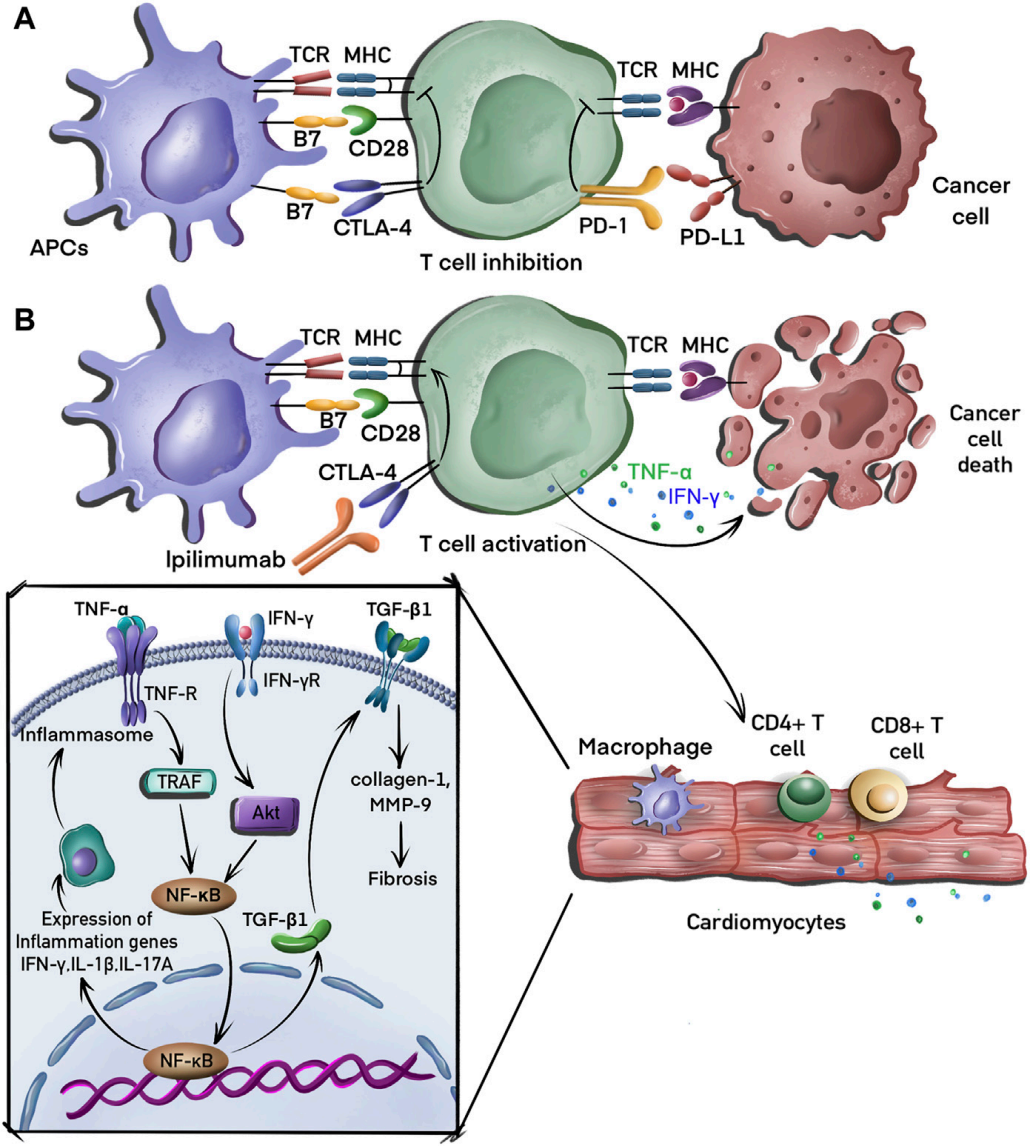
Molecular mechanisms of tumor evasion and cardiotoxicity induced by CTLA-4 blockade. **(A)** T cells recognize major histocompatibility complex (MHC) molecules on antigen presenting cells (APC), leading to the activation of these T cells, which then migrate to the tumor bed where they recognize cancer cells *via* the interaction between the MHC complex and T cell receptors (TCR) on the T cell. T cells receive inhibitory signals induced by the binding of CTLA-4 to B7 and PD-1 to PD-L1. **(B)** CTLA-4 inhibitor (ipilimumab) binds to CTLA-4 to activate T cells that eliminate tumor cell by secreting interferon-γ (IFN-γ) and tumor necrosis factor-alpha (TNF-α). CD4^+^ and CD8^+^ T cells also infiltrate the myocardial tissue, releasing TNF-α, which induces the production of proinflammatory cytokines, including IFN-γ, interleukin (IL)-2, and IL-17A *via* the activation of the nuclear factor-kappa B (NF-κB) signaling pathway. IFN-γ can also induce fibrosis by promoting the expression of transforming growth factor-beta 1 (TGF-β1).

CTLA-4 is expressed almost exclusively on T cells and binds to B7 molecules on antigen presenting cells (APCs) to promote tumor growth by inhibiting T cell immune response. Anti-CTLA-4 antibodies enhance immune responses and suppress neoantigen expression by activating the binding of CD28 to B7 and that of T cell receptor to major histocompatibility complex molecules, resulting in tumor cell elimination (Hu et al., 2019). The multiple processes involved in the mechanism of CTLA-4 blockade-associated cardiotoxicity, including fibrosis and inflammation, are summarized in Figure 1.

### 2.1 Inflammation

In a preclinical mouse model, CTLA-4 knockout induced T cell and macrophage infiltration in myocardial tissues that destroyed cardiomyocytes, leading to metabolic failure with high mortality (Wei et al., 2021). The activation of T cells and macrophages induces a proinflammatory phenotype in cardiac and vascular tissues by increasing the expression of nuclear factor-kappa B (NF-κB), the NLRP3 inflammasome, and MyD88 (Quagliariello et al., 2022). The blockade of CTLA-4 in cardiomyocytes also led to an increased levels of proinflammatory cytokines, including those of interferon-gamma (IFN-γ), tumor necrosis factor-alpha (TNF-α), interleukin (IL)-2, and IL-17A in the myocardium (Han et al., 2012). Although the underlying mechanism is unclear, it is hypothesized that CTLA-4 blockade leads to the differentiation of inflammatory effector CD4^+^ T cells that migrate back to the heart and increase the expression of these proinflammatory cytokines (Lichtman, 2013). Finally, CTLA-4 blockade therapy induces endothelial activation to increase the expression of intercellular adhesion molecule 1 (ICAM1) in the aortic endothelium. As the ligand for β2-integrin molecules, ICAM1 plays an important role in inflammation (Poels et al., 2020). The reduction of ICAM1 expression has been shown to decrease cardiac inflammation and ameliorate systolic dysfunction during pressure overload (Yoshida et al., 2015).

### 2.2 Fibrosis

Several clinical cases of interstitial fibrosis in myocardial tissue have been identified in endomyocardial biopsies after CTLA-4 inhibitor treatment (Heinzerling et al., 2016); however, the underlying mechanism is unknown. A preclinical study showed that the depletion of T cells enhances silica-induced fibrosis by increasing the secretion of transforming growth factor-beta 1 (TGF-β1), which is associated with the suppression of CTLA-4 expression (Liu et al., 2010). CTLA-4 blocking agents also increase myocardial fibrosis by modulating the expression of galectin-3, collagen 1, and matrix metalloproteinase 9 (MMP-9) (Quagliariello et al., 2022). By contrast, a recent study demonstrated that CTLA4-Ig-mediated immunosuppression limits subepithelial fibrosis (Khan et al., 2022). Further studies are required to elucidate the regulatory mechanism of CTLA-4 inhibitors.

## 3 Molecular mechanisms of cardiotoxicity induced by PD-1/PD-L1 blockade

**FIGURE 2:**
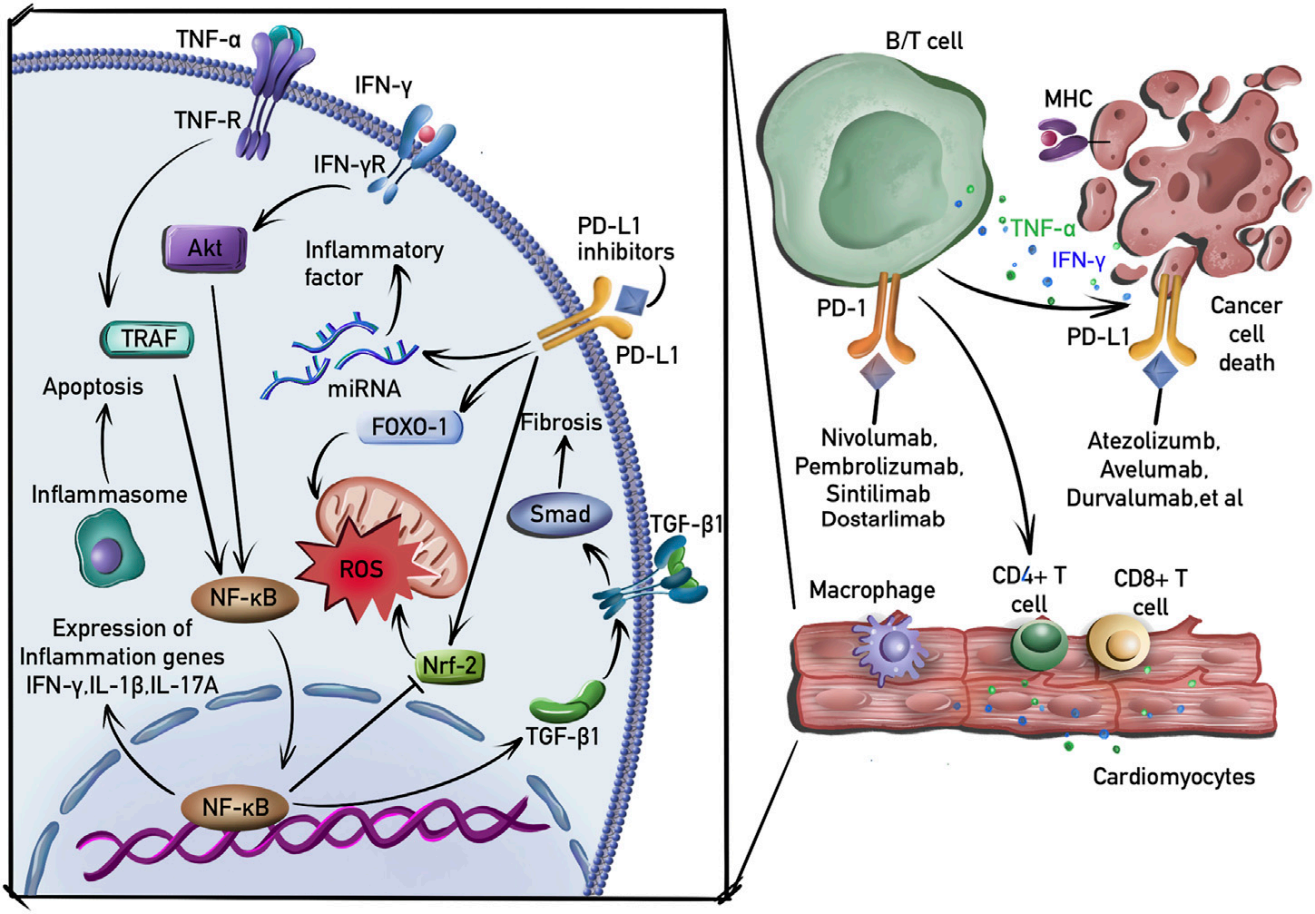
Molecular mechanisms of cardiotoxicity induced by PD-1/PD-L1 blockade. B and T cells are activated by PD-1 or PD-L1 inhibitors. Macrophages, CD4^+^ and CD4^+^ T cells then infiltrate the myocardial tissue. In addition to the induction of inflammation and fibrosis by activating NF-κB and TGF-β1 signaling pathway, PDL-1 inhibitors induce the production of ROS by suppressing nuclear factor erythroid 2-related factor-2 (Nrf-2) and upregulating transcriptional factor forkhead box-1 (FOXO-1). PD-1 inhibitors also activate macrophages and induce the expression of proinflammatory cytokines by regulating the expression of miRNAs.

PD-1 and PD-L1 are important targets expressed on most immune cells, including T cells and B cells. Tumor neoantigens released by cancer cells are captured and presented to T cells by APCs. However, PD-L1, which is expressed at high levels on cancer cells, binds to PD-1 on T cells, resulting in inhibitory checkpoint signaling that inhibits their activation, thereby allowing unregulated tumor growth. PD-1 blocking antibodies suppress the interaction of PD-1 with PD-L1, resulting in enhanced T cell cytotoxicity and ultimately in tumor cell elimination (Varricchi et al., 2017). The mechanisms considered to play an important role in cardiotoxicity induced by PD-1/PD-L1 blockade are presented in Figure 2.

### 3.1 Inflammation

It has been reported that the cardioprotective effects of the PD-1/PD-L1 interaction are mediated by suppressing the production of inflammatory factors and that the disturbance of this interaction neutralizes this protective effect to cause cardiotoxicity (Seko et al., 2007). The inhibition of the PD-1/PD-L1 interaction in an animal model has been shown to induce the expression of several proinflammatory factors, including TNF-α and IL-1β, leading to cardiomyocyte apoptosis (Chen et al., 2022). PD-1/PD-L1 blockade also induces cardiac inflammation by increasing the expression of IFN-γ and promoting cardiomyocyte apoptosis *via* a mechanism that involves the reduction of p-Akt expression (Tay et al., 2020). In addition, this inflammatory cardiotoxicity was associated with the infiltration of CD4^+^/CD8^+^ T cells (Ji et al., 2019). Furthermore, PD-L1 blockade reduced B-cell production of the anti-inflammatory cytokine IL-10, thereby limiting inflammatory responses (Wang and Tan, 2020).

Inflammation is a key process in the development of atherosclerosis, which is characterized by the accumulation of lipids and IFN-γ-producing T cells in atherosclerotic plaques (Buono et al., 2005). The stimulation of the PD-1 pathway delayed atherosclerosis progression by decreasing the frequency of atherogenic IFN-γ T cells (Grievink et al., 2021). However, PD-1/PD-L1 blockade also accelerated the progression of atherosclerosis by inducing a predominantly T cell-driven inflammation (Sun et al., 2020).

### 3.2 Fibrosis

PD-1^(−/−)^ mice develop atrial myocardial fibrosis (Fu et al., 2013). Although the underlying mechanism is not yet known, one clinical study showed that the concentrations of TGF-β1 were increased in patients with lower expression of PD-1 (Zhang et al., 2022c). In addition, decreased PD-1/PD-L1 expression was correlated with decreased TGF-β1 in a previous animal study (Zhang et al., 2015), whereas increased PD-1 expression induced TGF-β1 expression (Celada et al., 2018). It is well known that PD-1 blockade activates the proliferation of regulatory T cells (Kamada et al., 2019), which play an important role in the progression of fibrosis by activating TGF-β1 production (Kagawa et al., 2022). TGF-β1 is a primary factor that drives fibrosis *via* the activation of Smad- and non-Smad-based signaling pathways that produce myofibroblasts (Meng et al., 2016). PD-1 inhibitors also increase the expression of fibronectin, a major pro-fibrotic cytokine, in cardiomyocytes; fibronectin plays an important role in the fibrotic process (Quagliariello et al., 2022).

### 3.3 Oxidative stress

T cell activation can lead to increased reactive oxygen species (ROS) production, resulting in oxidative stress, which is involved in PD-1/PD-L1 blockade-induced cardiotoxicity (Aboelella et al., 2021). ROS production is increased in the atrial myocytes of PD-1^(−/−)^ mice (Fu et al., 2013). In addition, PD-1 blockade-induced oxidative stress is associated with an increase in IFN-γ-positive macrophages (Liu et al., 2021). IFN-γ induces ROS production by suppressing the PI3K/Akt/nuclear factor erythroid 2-related factor-2 (Nrf-2) pathway, resulting in reduced antioxidant activity (Niu et al., 2021). Furthermore, the inhibition of PD-1 results in the upregulated expression of transcriptional factor forkhead box-1 (FOXO-1), which promotes ROS production by limiting mitochondrial glucose oxidation and increasing lipid oxidation (Chistiakov et al., 2017).

### 3.4 Other mechanisms

Studies have demonstrated that ICI-induced cardiotoxicity is associated with the regulation of miRNA expression. For instance, PD-1 inhibitor treatment profoundly increased the expression of miR-34a-5p, which targets the serine/threonine-protein phosphatase 1 regulatory subunit 10, to induce cardiac senescence (Xia et al., 2020a). PD-1 inhibitors also induce macrophage polarization and the expression of some proinflammatory cytokines by activating miR-34a expression (Xia et al., 2020b).

## 4 Clinical evaluations of therapy against ICI-induced cardiotoxicity

ICI-associated myocarditis has a high rate of morbidity and mortality (Lyon et al., 2018). For instance, one observational study demonstrated that nearly 50% of all patients with ICI-induced myocarditis experienced a major adverse cardiac event, such as cardiovascular death, cardiogenic shock, or complete heart block (Mahmood et al., 2018b). Therefore, electrocardiogram (ECG), natriuretic peptides, and cardiac troponin measurements are recommended in all patients before starting ICI therapy according to the 2022 ESC Guidelines on cardio-oncology (Lyon et al., 2022). In addition, the management of ICI-induced cardiovascular toxicity should be performed depending on the severity of the cardiotoxicity, with termination of ICI in cancer patients with fulminant or nonfulminant ICI-associated myocarditis as the first consideration. Furthermore, such patients should be hospitalized and allocated a level 2 or 3 bed with continuous ECG monitoring. For milder ICI-mediated cardiotoxicity, the treatment strategies include immunosuppression to alleviate inflammatory changes and supportive therapy to address cardiac complications (Hu et al., 2019). Clinical studies on the efficacy of drug therapies for ICI-induced cardiotoxicity are summarized in Supplementary Table S1.

### 4.1 Methylprednisolone

In one clinical study, the rate of ICI-associated myocarditis reached approximately 25%, although only 2 of 35 patients exhibited the symptoms of a major adverse cardiac event after receiving 1,000 mg methylprednisolone (Mahmood et al., 2018b). Methylprednisolone is a common steroid that exerts rapid immunosuppressive effects by suppressing T cell function. Despite many case reports on these effects, a large randomized controlled trial is required to assess the efficacy of methylprednisolone in ameliorating cardiotoxicity induced by ICIs (Supplementary Table S1).

Sintilimab, a PD-1-specific monoclonal antibody, has been reported in numerous clinical studies and is widely used to treat multiple tumor types. Several case reports on the therapeutic efficacy of methylprednisolone in sintilimab-induced myocarditis and cardiac injury have been published (Xing et al., 2020; Bi et al., 2021; Lin et al., 2022); however, some patients who received sintilimab did not survive even after methylprednisolone treatment (Chen et al., 2021b).

Pembrolizumab is another PD-1 inhibitor approved by the US FDA in 2014 and has been used in many counties (Szuchan et al., 2020; Arangalage et al., 2021; Cao et al., 2021; Nguyen et al., 2022; Su et al., 2022; Yang et al., 2022). In cases of pembrolizumab-induced cardiotoxicity, patients present with the clinical symptoms of chest pain, shortness of breath, and dyspnea after treatment, with dramatically increased levels of myocardial injury markers, which decline to normal levels after methylprednisolone treatment (Supplementary Table S1). Methylprednisolone has been shown to ameliorate the symptoms of myocarditis and acute heart failure induced by other PD-1 inhibitors, including camrelizumab, nivolumab, tislelizumab, and toripalimab (Monge et al., 2018; Fazel and Jedlowski, 2019; Bai et al., 2021; Hu et al., 2021; Jespersen et al., 2021; Komatsu et al., 2021; Luo et al., 2021; Wang et al., 2021; Zhang et al., 2022a). These patients commonly exhibit the symptoms of chest pain and shortness of breath approximately 1 or 2 weeks after PD-1 inhibitor treatment. Methylprednisolone usually alleviates these symptoms and reduces the myocardial injury markers in serum; however, some patients still die owing to cardiac dysfunction and other reasons (Monge et al., 2018; Jespersen et al., 2021; Komatsu et al., 2021; Wang et al., 2021; Zhang et al., 2022a). Therefore, Methylprednisolone therapy requires optimization. Finally, methylprednisolone has been shown to suppress myocarditis induced by the CTLA-4 inhibitor ipilimumab (Fazel and Jedlowski, 2019).

### 4.2 Prednisone

Prednisone is another glucocorticoid steroid widely used for the treatment of ICI-induced myocarditis (Supplementary Table S1). Compared with receiving intravenous methylprednisolone, the effective time of oral prednisone is relatively short and dependent on the metabolic activation of the liver. In the clinic, prednisone is preferred for the treatment of patients with moderate immune responses after methylprednisolone treatment. Prednisone combined with another immunosuppressive agent usually provides benefit in patients with cancer (Nasr et al., 2018; Yamaguchi et al., 2018; Agrawal et al., 2019; Guo et al., 2020; von Itzstein et al., 2020; Luo et al., 2021), although some patients do not survive (Konstantina et al., 2019). Furthermore, one case report showed that prednisone alone improved cardiac function in a patient with ICI-induced myocarditis (Miyauchi et al., 2021).

### 4.3 Infliximab

As some patients cannot tolerate high-dose corticosteroids, other immunosuppressive drugs, such as infliximab, can be regarded as second-line therapies (Hu et al., 2019). The monoclonal antibody infliximab suppresses inflammatory responses by specifically binding and neutralizing TNF-α. Several case reports have described the ability of infliximab treatment to successfully ameliorate ICI-induced myocardial injury (Supplementary Table S1). In some cases, infliximab treatment could eliminated arrhythmias and cardiac dysfunction (Martinez-Calle et al., 2018; Padegimas et al., 2019). However, one retrospective study demonstrated that patients treated with infliximab were those with persistent evidence of cardiac dysfunction such as heart failure and ventricular tachycardia and that two of the four patients died from septic shock 2–3 months after the initial treatment (Zhang et al., 2021). Furthermore, in one case report, methylprednisolone combined with a high dosage of infliximab (10 mg/kg) did not improve the condition of a patient who ultimately died in the hospital (Gallegos et al., 2019). Therefore, for patients with symptoms of heart failure, a lower dose of infliximab (5 mg/kg) may be safer (Chung et al., 2003).

### 4.4 Immunoglobulins

Immunoglobulins, which are widely used to treat fulminant myocarditis and acute inflammatory cardiomyopathy, function by suppressing the expression of inflammatory and proinflammatory cytokines at the mRNA level (Shioji et al., 2001). Indeed, in some cases, intravenous immunoglobulins successfully suppressed severe myocarditis induced by ICIs (Supplementary Table S1). Similar to the situation with infliximab, immunoglobulins are used as a second-line therapy if methylprednisolone or other drugs are ineffective in treating ICI-induced cardiotoxicity (Norwood et al., 2017; Nasr et al., 2018; Yamaguchi et al., 2018; Agrawal et al., 2019; Balanescu et al., 2020; Yanase et al., 2021; Yang et al., 2021). However, immunoglobulins also failed in some patients (Chen et al., 2018; Agrawal et al., 2019; Imai et al., 2019; Ono et al., 2022; Saishu et al., 2022). Although the reason for differences in the effectiveness of methylprednisolone in some cases is unclear, we hypothesize that these differences are caused by variations in the underlying mechanism of ICI-induced cardiotoxicity in the different types of cancers.

### 4.5 Anti-thymocyte globulin (ATG)

ATG is a polyclonal antibody that modulates immune responses by inducing T lymphocyte depletion and commonly used to prevent acute allograft rejection (Valdez-Ortiz et al., 2015). ATG has been reported to improve cardiac function impaired by ICIs (Supplementary Table S1). In one case, ATG temporarily improved cardiac function in a patient with ongoing shock and hypotension (Jain et al., 2018). In some patients, ATG has produced a favorable outcome when combined with other drugs (Tay et al., 2017; Itzhaki Ben Zadok et al., 2019). However, it does not produce beneficial effects in some patients, especially in those with melanoma (Agrawal et al., 2019; Arora et al., 2020) or who received chemotherapy/radiotherapy prior to ATG treatment (McDowall et al., 2019). Therefore, further clinical investigations are required to fully elucidate the mechanism underlying the protective effects of ATG against ICI-cardiotoxicity and identify patients who are likely to benefit.

### 4.6 Mycophenolate mofetil

Similar to ATG, mycophenolate mofetil is used to prevent acute allograft rejection by inhibiting the proliferation of T and B lymphocytes. In some case reports, mycophenolate mofetil combined with a high dosage of methylprednisolone successfully suppressed fulminant immune-mediated myocarditis in patients with advanced endometrial cancer and melanoma (Mahmood et al., 2018a; Esfahani et al., 2019; Liu et al., 2020). Interestingly, one study showed that mycophenolate mofetil alone could rescue cardiac function impaired by PD-1 and CTL4 inhibitors (Reddy et al., 2017). However, in another case, this drug therapy decreased the levels of a cardiac injury biomarker, although the patient died because of respiratory failure (Yogasundaram et al., 2021). Moreover, one patient who received 10-month chemotherapy treatment with mycophenolate mofetil died (Konstantina et al., 2019).

### 4.7 Alemtuzumab

Alemtuzumab is a monoclonal antibody that binds to CD52, a protein present on the surface of multiple immune cells. One case report showed that alemtuzumab was used as an adjuvant medication for a patient with melanoma who suffered myocarditis for PD-1 therapy; the treatment gradually reduced cardiac biomarkers (Esfahani et al., 2019).

### 4.8 Abatacept

Abatacept is an agonist of CTLA-4 that alleviates nivolumab-induced myocarditis in lung cancer treatment (Salem et al., 2019) as well as pembrolizumab-induced myocarditis in melanoma treatment (Liu et al., 2020).

### 4.9 Tocilizumab

Tocilizumab is an IL-6 receptor inhibitor that plays an important role in ICI-associated myocarditis. One case report showed that intravenous tocilizumab was administered for one patient with lung cancer who developed myocarditis after nivolumab and ipilimumab treatments which was attenaued by Tocilizumab (Doms et al., 2020).

### 4.10 Plasmapheresis

Plasmapheresis, which is commonly used for therapeutic plasma exchange, is now used as a third-line approach for treating ICI-induced myocarditis owing to its ability to rapidly remove circulating immune complexes from the blood of the patient (Patel et al., 2021). Plasmapheresis also successfully suppresses immune responses induced by ICIs in combination with other drugs (Frigeri et al., 2018; Nasr et al., 2018; Liu et al., 2020; Yanase et al., 2021; Yogasundaram et al., 2021).

## 5 Therapeutic mechanisms of other agents used for ICI-induced cardiotoxicity

There is now a large body of evidence showing that chemical substances and natural products from Chinese herbs can attenuate cardiotoxicity induced by ICIs (Table 1).

**TABLE 1:** The mechanisms by which some chemical substances and natural products treat ICIs-induced cardiotoxicity.

Name	Type of study	Treatment method		Treatment Duration	Targets or pathways	Outcome	References
		Experiment group	Negative control group				
SRK-181	PD-1 antibody injection (10 mg/kg twice a week for 9 doses) in MBT-2 tumor xenografts of nude mice	SRK-181-mIgG1 (10 mg/kg once a week for five doses)	The same volume of control mIgG1	4 weeks	Inhibit TGF-β1 signaling pathway	Increased survival rate, decreased inflammatory cell infiltration in myocardium, eliminate cardiac valvopathy	Martin et al. (2020)
TNF-α antibody	PD-1 antibody injection (250 μg every second day for 9 doses) in melanoma tumor xenografts of C57 mice	TNF-α antibody (every second day for four does)	The same volume of control IgG1	2 weeks	Block TNF-αsignaling pathway	Improved LVEF	Michel et al. (2022)
IL-17A antibody	PD-1 antibody injection (200 μg every second day for 6 doses) in mice	IL-17A antibody (200 μg every second day for 6 doses)	The same volume of control IgG1	2 weeks	Block IL-17A signaling pathway	Improved LVEF	Gergely et al. (2022)
Butyrate produced by Prevotellaceae	PD-1/PD-L1 inhibitor (BMS-1) injection (10 mg/kg, every 2 days for 6 doses) in C57 mice	Sodium butyrate (1 g/kg/day, orally administration)	The same volume of saline by gavage	2 weeks	Suppress the expression of proliferator-activated receptor α (PPARα) and cytochrome P450 (CYP) 4X1	Prevented NF-κB activation and decreased cardiomyocyte apoptosis	Chen et al. (2022)
Levothyroxine	PD-L1 antibody injection (10 μg/g, every 1 week for 6 doses) in C57 mice	Levothyroxine (0.25 μg/g, every 1 week for 6 doses, i.p.)	The same volume of vehicle, i.p	3 weeks	Block NF-κB signaling pathway	Improved LVEF and decreased the expression of	Chen et al. (2021c)
Crocin	PD-1 antibody injection (5 mg/kg every 2 days for 5 times)	Crocin (25, 50 mg/kg/day, i.p.)	The same volume of vehicle, i.p	6 weeks	No mention	Attenuated the mortality rate and suppress inflammatory cell infiltration	Zhang et al. (2022b)

i.p., intraperitoneally; LVEF, Left ventricular ejection fraction.

Immunoglobulins that bind to CTLA-4 and PD1/PD-L1 have been shown to ameliorate myocarditis (Wei et al., 2021). The CTLA-4-Ig abatacept attenuates ICI-associated myocarditis by blocking T cell stimulation through its ability to bind to B7 ligands. Abatacept treatment significantly decreased myocardial immune infiltrates and reduced mortality in PD-1^(−/−)^ mice (Wei et al., 2021). Abatacept has also been beneficial in some clinical cases of ICI-induced cardiotoxicity (Salem et al., 2019; Liu et al., 2020).

TGF-β1 plays an important role in fibrosis induced by CTLA-4 and PD-1/PD-L1 inhibitors (Liu et al., 2010; Kagawa et al., 2022). SRK-181, a highly selective antibody inhibitor of TGF-β1, has been shown to increase intratumoral CD8^+^ T cells and decrease the frequency of immunosuppressive myeloid cells after anti-PD-1 treatment in an animal model (Martin et al., 2020). In addition, the blockade of TGF-β1 using a specific monoclonal antibody reduced tumor sizes (Terabe et al., 2017). Therefore, the combination of anti-TGF-β1 and anti-PD-1 has been implicated as a promising therapy for ICI-induced cardiotoxicity.

TNF-α has been predicted to mediate the cardiotoxic effects of ICI therapy. Increased cardiac TNF-α level was detected in tumor-bearing mice receiving anti-PD1 therapy, and TNF-α blockade improved the ejection fraction by enhancing the expression of lymphocyte-activation gene 3 and T cell immunoglobulin and mucin-domain containing-3 (TIM3), thereby enhancing the efficacy of anti-PD1 therapy (Michel et al., 2022).

IL-17A, which is an inflammatory cytokine produced primarily by Th17 cells, is activated by anti-PD1 treatment (Dulos et al., 2012). Furthermore, IL-17A was found to be associated with ventricular dilation and dysfunction in a mouse model of autoimmune myocarditis (Baldeviano et al., 2010). A recent study revealed that treatment with anti-IL17A antibody prevented the decline in ejection fraction and alleviated the cardiac dysfunction induced by PD-1 inhibitor (Gergely et al., 2022).

PD-1/PD-L1 inhibitors lead to gut microbiota dysbiosis, including the depletion of prevotellaceae and low production of microbial butyrate. Prevotellaceae enrichment or butyrate supplementation alleviated PD-1/PD-L1 inhibitor-induced myocardial enzyme leakage and myocardial apoptosis (Chen et al., 2022). These effects were associated with the upregulation of PPARα–CYP4X1 axis and downregulation of IL-1β and TNF-α in colonic macrophages. These findings provide a notable target for improving the safety of immunotherapy.

Interestingly, one study showed that levothyroxine rescued mice from ICI-caused mortality (Chen et al., 2021c). Cardiac function was significantly impaired at the early stage after PD-L1 inhibitor injection, with decreased cardiac ejection function and cardiac electrical conduction as well as a reduction in the body temperature, and these symptoms were effectively relieved by levothyroxine. However, the underlying mechanism of this effect remains to be elucidated.

Crocin, which is a major apo-carotenoid derived from saffron, partially reversed ICI-related myocarditis by improving cardiac function and alleviating myocardial injury. Mechanistically, it was shown that crocin inhibits theNLRP3 inflammasome and cleaved gasdermin D (GSDMD) and IL-1β (Zhang et al., 2022b). Therefore, we believe that Chinese herbs should be considered as promising therapeutic agents for the treatment of cardiotoxicity induced by ICIs.

## 6 Discussion

The pathogenesis of ICI-induced myocarditis is complex as multiple signaling pathways are activated by the infiltration of B and T lymphocytes as well as macrophages. Current immunosuppressive agents function mainly by suppressing B and T lymphocyte function or inhibiting inflammatory responses; however, according to the case reports reviewed here (Supplementary Table S1), approximately 50% of patients still do not survive such treatments. Although there is no widely accepted theory to account for the underlying mechanisms, we believe that once the symptoms of myocarditis present, the downstream signaling pathways, including inflammation (Han et al., 2012; Ji et al., 2019), fibrosis (Quagliariello et al., 2022), oxidative stress (Liu et al., 2021), and even apoptosis (Chen et al., 2022), are all activated. However, myocyte fibrosis/necrosis is a common histopathologic finding in patients with cancer treated with ICIs (Touat et al., 2018; Gallegos et al., 2019; Padegimas et al., 2019; Quagliariello et al., 2019); therefore, the suppression of B and T lymphocytes alone may not stop the immune response. Some preclinical studies showed that the blockade of signaling pathways significantly increased the survival rate and improved the cardiac function of patients with ICI-induced cytotoxicity (Supplementary Table S1). Therefore, we hypothesize that an alternative therapy combined with these immunosuppressive agents and blockade inhibitors will prove beneficial; however, this remains to be confirmed in clinical trials.

In this review, we reviewed 71 patients with cancer (40 male and 31 female) treated with anti-PD-1/PD-L1 and anti-CTLA-4 inhibitors. These patients were diagnosed with metastatic melanoma (n = 22), lung cancer (n = 10), thymoma (9), renal cell carcinoma (n = 6), esophagogastric carcinoma (n = 3), breast cancer (n = 2), gastric cancer (n = 2), myelodysplastic syndrome (n = 2), prostate cancer (n = 2), squamous cell cancer (n = 2), urothelial cancer (n = 2), advanced endometrial cancer (n = 1), chordoma (n = 1), colonic carcinoma (n = 1), glioblastoma (n = 1), laryngeal cancer (n = 1), lymphoma (n = 1), mesothelioma (n = 1), myeloma (n = 1), and ovarian adenocarcinoma (n = 1). The common symptoms included chest pain, chest tightness, dyspnea (shortness of breath), fatigue, muscle weakness, and occasional fever (Yamaguchi et al., 2018; Konstantina et al., 2019; Balanescu et al., 2020; Guo et al., 2020; Ono et al., 2022; Yang et al., 2022). Two patients even presented with a rash in the extremities or chest (Konstantina et al., 2019; Cao et al., 2021). All the patients were diagnosed with myocarditis *via* multiple techniques, including cardiac biomarkers in serum, ECG, echocardiography, cardiac magnetic resonance, or cardiac computed tomography (Supplementary Table S1).

After diagnosis, ICIs were stopped and immunosuppressive agents were immediately applied, including high-dose methylprednisolone, prednisone, infliximab, immunoglobulin, ATG, mycophenolate mofetil, alemtuzumab, abatacept, plasmapheresis, tocilizumab, rituximab, tacrolimus, or methotrexate (Supplementary Table S1). Of 41 patients treated initially with methylprednisolone, 25 (61%) were alive after subsequent immunosuppressive therapy (Supplementary Table S1), whereas of 16 patients who received methylprednisolone treatment alone, only 7 (43%) were alive with a decline in cardiac biomarkers and improved cardiac function (Xing et al., 2020; Bai et al., 2021; Bi et al., 2021; Hu et al., 2021; Lin et al., 2022; Su et al., 2022; Yang et al., 2022). Thus, it appears that a combination of multiple drugs can promote overall survival, suggesting that when the effects of methylprednisolone treatment alone are unsatisfactory, outcomes are improved by adding other immunosuppressive agents. Furthermore, six patients were treated initially with prednisone, followed by other immunosuppressive agents; three (50%) were alive after immunosuppressive therapy (Nasr et al., 2018; Yamaguchi et al., 2018; Agrawal et al., 2019) and two patients were alive after receiving prednisone alone (Guo et al., 2020; Miyauchi et al., 2021).

Some other organ systems are affected by ICIs, including the gastrointestinal tract, skin, lung, and liver (Weber et al., 2017), with reports of patient deaths caused by respiratory failure (Arora et al., 2020; Komatsu et al., 2021; Saishu et al., 2022), pulmonary encephalopathy (Chen et al., 2021b), infection (Konstantina et al., 2019; Padegimas et al., 2019), and even multiorgan failure (Chen et al., 2018; Agrawal et al., 2019). Therefore, the effects of ICIs on the function of other organs should be considered.

## Conclusion

Herein, we reviewed the case reports of ICI-induced myocarditis, which is a serious adverse event with a high fatality rate. Although one retrospective study demonstrated that the 1-year survival was significantly higher in patients with cancer who received ICI-based immunosuppressive therapy (Joseph et al., 2020), this strategy remains to be optimized.

The limitations of our review should be noted. First, the majority of the clinical information was based on case reports and there is a lack of large-scale, multicenter, randomized, and controlled clinical trials on the use of immunosuppressive agents to treat ICIs-induced cardiotoxicity. Second, there was wide variation in the characteristics of the referred cases, including age, tumor type, and stage. In addition, follow-up after discharge was not performed in some cases, whereas others were followed-up for several months. Third, a large proportion of the published cases focused on suppressing the immune response in the myocardium, although the function of other organs was also affected. Finally, there were no tumor controls without ICI therapy in these published cases. To overcome these limitations, we believe that future research studies should be focused on at least two aspects. First, large-scale, multicenter, and randomized controlled trials are urgently required to investigate the efficacy of different immunosuppressive agents on different tumor types. Second, products derived from Chinese herbs offer several outstanding advantages that indicate their promise as a therapeutic option. Since, natural products usually have few side effects compared with synthetic drugs. In addition, natural products also have multiple pharmacological activities, providing enhanced beneficial effects owing to the involvement of multiple signaling pathways in the process of ICI-induced myocarditis.
