# Supplementary material for: Drug therapy for myocarditis induced by immune checkpoint inhibitors
Source: Front Pharmacol. 2023 May 25;14:1161243. doi: 10.3389/fphar.2023.1161243 (PMC10248045; doi:10.3389/fphar.2023.1161243)
Supplement: Supplementary file 1 [file Table1.doc]

**TABLE 1** Immunosuppressive agents used in the included cases

| **Research**  **design** | **Country** | **Sample size** | **Age/Sex** | **Cancer type** | **ICIs therapy/**  **After initial treatment** | **Symptoms/Cardiotoxicity/Diagnostic method** | **Treatment method** | **Outcome** | **Reference** |
| --- | --- | --- | --- | --- | --- | --- | --- | --- | --- |
| **Case report** | China | 1 | 68/M | Non-small cell lung cancer | Sintilimab/6 days | A productive cough and progressive dysphagia/Myocarditis/ECG; Coronary angiography | Methylprednisolone (80 mg/day for 4 days, then decreased to 40 mg/d) | cTnT, CK-MB, CK and BNP were reduced/Alive | Bi et al., 2021 |
| **Case report** | China | 1 | 66/M | Lung adenocarcinoma | Sintilimab/12 days | Shortness of breath and progressive muscle weakness/ Myocarditis (cTnT: 0.916 ng/ml CK:11919 U/L)/CT | Methylprednisolone (2 g/day for 5 days and immunoglobulin (400 mg/kg/d for 5 days) | cTnT and CK were reduced/ALive | Xing et al., 2020 |
| **Case report** | China | 1 | 66/M | Lung adenocarcinoma | Sintilimab/3 weeks | Chest pain, shortness of breath/Myocarditis (cTnT: 9.4 ng/ml; CK: 922 IU/L; CK-MB: 109 ng/ml; NT-proBNP:8290 pg/ml; LEVF: 35%)/ ECG; Echocardiography; CMR | Methylprednisolone (2 g/day for 7 days and gradually decreased) and immunoglobulin (0.4g/kg/d) | cTnT, CK, CK-MB and NT-proBNP were reduced, LEVF was increased /Alive | Lin et al., 2022 |
| **Case report** | China | 2 | 77/M  69/F | Chordoma  Non-small cell lung cancer | Sintilimab + Anlotinib/3 weeks  Bevacizumab + camrelizumab/20 days | Chest tightness, shortness of breath/ Myocarditis (cTnT: 1.29 ng/ml; NT-proBNP:581.8 ng/L; CK-MB:140.7 ng/ml; CK: 706 U/L)/ECG; PET-MRI; Echocardiogram  Sinus tachycardia, atrial premature beats, atrial tachycardia/Myocarditis (cTnT: 0.952 ng/ml; NT-proBNP:321 ng/L; CK-MB:81.8 ng/ml)/ ECG; Echocardiogram; | Methylprednisolone (480 mg/day for 5 days and gradually reduced to 40 mg/day)  Methylprednisolone (480 mg/day for 5 days and decreased to 120 mg/d in the next two weeks) | CK and cTnT levels was declined/Dead  cTnT and CK-MB were reduced/Dead | Chen et al., 2021b |
| **Case report** | China | 1 | 51/F | Breast cancer | Pembrolizumab/ 3 days | High fever, mild dyspnea, and systemic rash/Myocarditis (BNP: 1991 pg/mL)/CT | Methylprednisolone (80 mg/day for 7 days and gradually decreased to 20 mg/kg) | Myocardial injury markers were reduced to normal levels/Alive | Yang et al., 2022 |
| **Case report** | China | 1 | 80/F | Squamous cell cancer | Pembrolizumab and nimotuzumab/3 weeks | Palpitation, faintness, and general fatigue /Myocarditis (hsTnI: 1026.5 pg/mL; CK: 510 U/L; CK-MB: 39.8 U/L; BNP: 2171 pg/mL)/ECG; Echocardiography | Methylprednisolone (1 g/day for 7 days and gradually decreased) | The level of hsTnI, BNP, and CK were gradually reduced to normal/Alive | Su et al., 2022 |
| **Case report** | France | 1 | 25/M | Thymoma | Pembrolizumab/14 days | Chest pain, subtle myalgia/Myocarditis, heart failure (TnT: 3000 ng/L; CK: 3714 IU/L; LVEF: 20%)/MRI; CT; EMB | Methylprednisolone (1 g/day for 3 days and gradually decreased); mycophenolate-mofetil (1 g for 2 days), abatacept (20mg/kg for 5 dosage and ruxolitinib (30 mg/day） | TnT and LVEF was restored to normal/Alive | Nguyen et al., 2022 |
| **Case report** | China | 1 | 69/M | Esophagogastric carcinoma | Pembrolizumab+ Oxaliplatin/2 weeks | Limb weakness and shortness of breath/ Myocarditis, heart failure (CK: 6016 U/L; CK-MB: 128.4 ng/Ml; cTnT: 2.83 ng/mL; NT-proBNP: 3644 pg/mL; LVEF: 30%) /ECG; Echocardiography | Methylprednisolone (500 mg/day for 3 days and gradually decreased to 30 mg/day), immunoglobulin (20 g) | Myocardial enzymes showed a downward trend and LVEF increased to 45%/Alive | Cao et al., 2021 |
| **Case report** | USA | 1 | 70/M | Thymoma | Pembrolizumab/ 16 days | Exertional dyspnoea, orthopnoea, and weakness/Myocarditis (TnT: 10.50 ng/L; CK: 1667 U/L; CK-MB: 107 U/L) /CT; ECG; EMB | Methylprednisolone (1 g /day for 3 days) and prednisone (1 mg/kg/day) | Myocardial enzymes decrease/Alive | Szuchan et al., 2020 |
| **Case report** | Switzerland | 1 | 61/F | Melanoma | Pembrolizumab/3 months | Chest pain/Myocarditis (TnT: 266 ng/L; CK: 158 U/L)/ECG; CT; Echocardiography; PET | Methylprednisolone (1 g /day for 3 days) and trametinib (2 mg/day) | Cardiac function is improved/Alive | Arangalage et al., 2021 |
| **Case report** | China | 1 | 69/M | Esophageal carcinoma | Camrelizumab combined with chemotherapy/3 weeks | Palpitations/Myocarditis (CK: 3503.1 U/L; CK-MB: 178.7 U/L; cTnI: 0.35 ng/mL) /CT; ECG | Methylprednisolone (120 mg/day for 5 days) and gradually decreased | The levels of biomarkers of myocardial injury declined/Alive | Bai et al., 2021 |
| **Case report** | China | 1 | 69/F | Thymoma | Camrelizumab/ 11 days | Dyspnea, fatigue, muscle weakness/ Myocarditis (TnI: 13.94 ng/mL; CK-MB 213.70 ng/mL; CK 8811.40 U/L; NT-proBNP: 1169.5 pg/ml) /ECG; MRI; | Methylprednisolone (80 mg/day), Polyene phosphatidylcholine (465 mg/day), reduced glutathione (1.8 g/day) for 1 day; Methylprednisolone (1 g/day) and β-blocker for 2 days | TnI and CK were reduced/Dead | Zhang et al., 2022a |
| **Case report** | China | 1 | 66/M | Gastric cancer | Nivolumab/24 days | Dizziness and difficulty breathing/ Myocarditis (CPK: 8903 IU/L; CK-MB: 289 IU/L; TnI: 16256 pg/mL)/CT; ECG; EMB | Methylprednisolone (1 g/  day for 7 days) followed with immunoglobulins (22.5 g/day) | Dead | Komatsu et al., 2021 |
| **Case report** | USA | 1 | 79/M | Prostate cancer | Nivolumab/8 weeks | Blurred vision and pain and stiffness in the upper back/Myocarditis (CK: 3200 U/L; CK-MB: 65.7 mcg/L; TnI: 0.209 ng/mL; ProBNP: 3066 pg/mL)/ ECG; Echocardiogram; MRI; | Methylprednisolone (1 mg/kg/day) and gradually decreased | Cardiac enzymes are normalized/ Dead | Monge et al., 2018 |
| **Case report** | China | 1 | 56/M | Colonic carcinoma | Nivolumab/1.5 months | Chest tightness and pain CT/Myocarditis (NT-proBNP: 180.40 ng/L; CK-MB: 8.99 ng/mL; TnT:0.23 ng/mL; LVEF: 50%)/ECG | Methylprednisolone (160 mg/day for 3 day) and immunoglobulin (25 g/day for 5 days) | The biomarkers levels of myocardial injury declined/Dead | Wang et al., 2021 |
| **Case report** | USA | 1 | 78/F | Metastatic  melanoma | Nivolumab and Ipilimumab/5 days | Weakness and myalgias/ Myocarditis (CPK:9198 IU/L; TnT 8.57 ng/mL)/ MRI; | Methylprednisolone (1 mg/kg body for 3 days and gradually decreased), immunoglobulin (2 mg/kg for 2 doses) | Muscle weakness mildly improved/Alive | Fazel and Jedlowski, 2019 |
| **Case report** | Denmark | 1 | 57/M | Renal cell carcinoma | Nivolumab and Ipilimumab/12 days | Headache and myalgia/ Myocarditis (TnI 5760 ng/L; CPK 3510 U/L)/ ECG; CT; CMR; TTE | Methylprednisolone (1000 mg/kg/day for 2 days) followed with abatacept (500 mg every 2 weeks, for 5 doses) and Mycophenolate mofetil (1 g twice a day for 3 months) | TnI and CPK level decreased/Dead | Jespersen et al., 2021 |
| **Case report** | China | 1 | 66/M | Ureteral urothelial cancer | Tislelizumab/No mention | Chest tightness/Myocarditis (Hs-TnI: 9317 ng/L; CK-MB: 84.5 ng/ml; NT-proBNP: 291 pg/ml; CK: 4700 U/L)/ ECG; CT; CMR; Echocardiography | Methylprednisolone (1.5 g/kg/day for 3 days) | The biomarkers levels gradual recovery/Alive | Hu et al., 2021 |
| **Case report** | China | 1 | 47/F | Thymoma | Toripalimab/4 weeks | Myalgia and limb weakness/ Myocarditis (TnI:2.796 ng/ml; CK: 25200 U/L)/ECG; | Methylprednisolone (500 mg/day for 10 days); Prednisolone (60 mg/d for 4 weeks) | The laboratory markers were reduced/Alive | Luo et al., 2021 |
| **Case report** | Australia | 6 | 74/M  60/F  75/M  74/F  88/F  61/M | Metastatic  Melanoma  Metastatic  Melanoma  Metastatic  Melanoma  Metastatic  Melanoma  Metastatic  Melanoma  Metastatic  Melanoma | Cobimetinib， atezolizumab and pembrolizumab/ 14 days  Cobimetinib， atezolizumab and pembrolizumab/ 13 days  Pembrolizumab/ 20 days  Pembrolizumab/8 weeks  Pembrolizumab/3 weeks  Pembrolizumab/3 weeks | Fatigue, fevers, chills, anorexia, diarrhoea/Myocarditis (hs-TnT:77 ng/L; CK: 1165 U/L)/ECG; TTE  Nausea, vomiting, diarrhoea and mucositis/Myocarditis (CK:377 U/L; hs-TnT: 455 ng/L)/CMRI; TTE  Chest wall, omentum, and lymph nodes/ Myocarditis (CK:13025 U/L; hs-TnT: 2978 ng/L)/CMRI; TTE  Dyspnoea/ Myocarditis (CK:3769 U/L; hs-TnT: 501 ng/L; LVEF:35%)/ ECG CMRI; TTE;EMB  Dyspnoea/ Myocarditis (hs-TnT: 2146 ng/L; LVEF:18%) /TTE; CMRI； PET  Dyspnoea/ Myocarditis (LVEF:18%) / ECG; TTE; CMRI; PET | Methylprednisolone (2 mg/kg for 3 days) followed with prednisolone (2 mg/kg for 8 weeks)  Methylprednisolone (1 mg/kg for 3 days) followed with prednisolone (1 mg/kg for 6 weeks)  Methylprednisolone (1 g/day for 3 days) followed with prednisolone (1 mg/kg for 6 weeks)  Methylprednisolone (2 mg/kg for 4 days)， prednisolone(2 mg/kg for 6 weeks) and Mycophenolate mofetil( 2 g/day for 6 weeks)  Methylprednisolone (1 g/day for 4 days)， prednisolone(2 mg/day for 6 weeks)  Prednisolone (1 mg/day for 6 months) | CK and hs-TnT declined/Alive  hs-TnT and CK  normalized/Alive  CK and hs-TnT normalized/Alive  Cardiac enzymes were decline/Alive  Cardiac enzymes were decline/Dead  LVEF was improved/Alive | Guo et al., 2020 |
| **Case report** | USA | 5 | 73/M  64/M  89/M  65/F  67/M | Mesothelioma  Metastatic  Melanoma  Metastatic  Melanoma  Laryngeal cancer  Metastatic  Melanoma | Pembrolizumab/32 days  Pembrolizumab/28 days  Pembrolizumab/3 weeks  Nivolumab/6 days  Nivolumab/ 3 weeks | Dyspnea and fatigue/ Myocarditis (TnI: 8.3 ng/mL; CK: 6124 u/L; CK-MB: 8.3 ng/mL)/ECG  Fatigue, weakness, and myalgia/ Myocarditis (TnI: 0.78 ng/mL; CK:1681 IU/L; CK-MB: 159 ng/ml)/ECG; MRI  Dyspnea, weakness, and myalgias/ Myocarditis (CK:964 IU/L, CK-MB:  104.4 ng/mL, TnI 5.78 ng/mL)/ ECG  No mention/Myocarditis (CK-MB：2.2 ng/mL, TnI:0.12 ng/mL, and BNP 330 pg/mL) /ECG;MRI  Chest pain/ Myocarditis (TnI:0.31 ng/mL)/ECG; MRI | Prednisone (60 mg for 2 doses) combined with immunoglobulin (5 doses)  Prednisone (100 mg for 7 days) combined with immunoglobulin (5 doses)  Methylprednisolone(1g/day); prednisone (120 mg/day); ATG (125 mg/day)  Methylprednisolone (1 g/day for 2 days) following with ATG (3 doses)  Methylprednisolone (1 g/day for 3 days) following with infliximab | No improvement /Dead after 20 days  Improved/Alive  No improvement/Dead  No improvement/Dead  Clinical symptoms improved/Alive | Agrawal et al., 2019 |
| **Case report** | Japan | 1 | 60/M | Metastatic  Melanoma | Nivolumab/30 days | Fatigue and fever/ Myocarditis (TnT:2526 U/L; LVEF:15%)/ECG; Echocardiography; EMB | Prednisolone (1 g/day for 3 days) combined with immunoglobulin (50 g/day for 2 days) | Clinical symptoms improved/Alive | Yamaguchi et al., 2018 |
| **Case report** | France | 1 | 79/M | Gastric cancer | Pembrolizumab/1 weeks | Bilateral asymmetric ptosis/ Myocarditis (CPK:4893 ng/L; CPK-MB:128 U/L; TnI: 3476 ng/mL)/ MRI; ECG; EMB | Prednisone (2 mg/kg for 14  days), immunoglobulins (2 mg/kg for 4 days), plasmapheresis for 3 days and methotrexate (25 mg/m^2^) | CPK levels were decreasing/Alive | Nasr et al., 2018 |
| **Case report** | USA | 1 | 65/M | Non-small cell lung cancer | Durvalumab/1 month | Weakness, myalgias, diplopia, and chest tightness/ Myocarditis (CPK: 5144 U/L; hs-TnT: 296 ng/L)/ Electrocardiogram; ECG | Methylprednisolone (1 mg/kg) and prednisone 1 mg/kg for 37 days) | CPK and hs-TnT was decreased/Alive | von Itzstein et al., 2020 |
| **Case report** | Greece | 2 | 58/F  30/F | Thymoma  Thymoma | Pembrolizumab/ 1 weeks  Pembrolizumab/ 3 days | Fever and rash/ Myocarditis (LVEF:30%)/ECG; Echocardiography  Chest pain and muscle weakness/ Myocarditis/ECG; Echocardiography | Prednisolone (1 mg/kg for 2 days) following with β-blocker, antiarrhythmics, and mycophenolate mofetil  Prednisolone (2 mg/kg for 2 days), immunoglobulin (400 mg/kg for 5 days) and rituximab | No improvement/Dead  No improvement/Dead | Konstantina et al., 2019 |
| **Case report** | Japan | 1 | 71/M | Renal cell carcinoma | Nivolumab and Ipilimumab/12 days | Chest tightness and shortness of breath/Myocarditis (23488 U/L; TnT:100680 pg/mL; NT-proBNP: 15964 pg/mL)/ECG; MRI; Echocardiography; EMB | Prednisolone (60 mg/day for 6 days) and gradually decreased | The biomarkers levels of myocardial injury were decreased/Alive | Miyauchi et al., 2021 |
| **Case report** | USA | 2 | 53/F  62/F | Ovarian adenocarcinoma  Renal cell carcinoma | Pembrolizumab/4 days  Nivolumab/5 weeks | No mention/Myocarditis (TnT:0.659 ng/ml; NT-proBNP:815 pg/ml/; LVEF: 50%)/ ECG; Echocardiogram; CMR;  Chest tightness/ Myocarditis (TnT: 36.2 ng/ml; LVEF: 25%)/ECG; CMR; EMB | Methylprednisolone (1 mg/day for 3 days) continued with infliximab (5 mg/kg for 1 day)  Methylprednisolone (2 mg/day for 3 days) continued with infliximab (5 mg/kg for 1 day) | TnT and LVEF were normalized/Alive  LVEF back to 55%/Dead | Padegimas et al., 2019 |
| **Case report** | Spain | 1 | 67/F | Myeloma | Pembrolizumab/ 12 days | Dyspnea/ Myocarditis (hsTnT: 9.71 ng/mL; CK: 3689 UI/L; CK-MB 300 ng/mL)/ECG; Echocardiogram; | Methylprednisolone (1.5 mg/day for 1 days) infliximab (5mg/kg) | cardiac biomarkers were showed normal/Alive | Martinez-Calle et al., 2018 |
| **Case report** | USA | 1 | 47/F | Metastatic  Melanoma | Ipilimumab and nivolumab/ 13 days | Dyspnea/ Myocarditis (proBNP: 3797 pg/ml; LEVF: 26%/ECG; CMR; Echocardiogram | Methylprednisolone (500 mg for 5 days) continued with infliximab (10 mg/kg/day for 2 days) | No rescue/Dead | Gallegos et al., 2019 |
| **Case report** | USA | 1 | 49/F | Metastatic  Melanoma | Nivolumab and ipilimumab/2 weeks | Nausea/ Myocarditis (cTnI to 0.19 ng/mL; CK-MB: 6.3 ng/mL; CK: 335 U/L) /ECG; EMB; Echocardiogram; CMR | Methylprednisolone (125 mg/day for 3 days) continued with immunoglobulin (400 mg/kg/day for 2 days) | The levels of cTnI and CK-MB were normalize/Alive | Norwood et al., 2017 |
| **Case report** | Japan | 1 | 59/M | Renal cell carcinoma | Ipilimumab and nivolumab/21 days | Bilateral ptosis and malaise/ Myocarditis (TnI: 3.78 ng/mL; CPK: 8944 ng/mL; CPK-MB: 180 ng/mL) /Electrocardiogram; CT; ECG; EMB | Methylprednisolone (1 g/day for 3 days) following with immunoglobulin (400 mg/kg for 4 days) and plasmapheresis (4 cycle) | Cardiac biomarkers were improved/Alive | Yanase et al., 2021 |
| **Case report** | USA | 3 | 75/M  78/M  74/F | Myelodysplastic syndrome  Myelodysplastic syndrome  Metastatic melanoma | Ipilimumab, nivolumab, and azacytidine/33 days  Ipilimumab and azacytidine/33 days  Ipilimumab and evofosfamide/28 days | Fever, cough, dyspnea/ Myocarditis (cTnI: 2.94 ng/ml; BNP: 1191 pg/mL)/ECG; CT;  Fever and pneumonia/Myocarditis (cTnI: 12.45 ng/ml; BNP: 1988 pg/mL; LVEF: 55%)/ECG; CT; EMB; Echocardiography  Dyspnea and fever/Myocarditis (cTnI: 3.72 ng/ml; BNP: 193 pg/mL; LVEF: 55%)/ECG; CT; Echocardiography; EMB | Immunoglobulin (1 mg/kg for 4 days) and rosuvastatin (20 mg for day)  Immunoglobulin, colchicine, and atorvastatin  Immunoglobulin, colchicine, rosuvastatin, and hydroxychloroquine | Improved/Alive  Cardiac function normalized/Alive  Improved/Alive | Balanescu et al., 2020 |
| **Case report** | China | 1 | 33/M | Thymoma | Sintilimab/24 days | Dyspnea, palpitation, and muscle weakness/ Myocarditis (CK:1324 IU/L; NT-proBNP: 154 ng/ml; TnT: 69 ng/ml)/ ECG; echocardiography | Methylprednisolone (2 mg/kg/d), immunoglobulin (20g/d for 5 days), and pyridostigmine (180 mg/day) | The symptoms significantly improved/Alive | Yang et al., 2021 |
| **Case report** | Japan | 1 | 70/M | Squamous cell carcinoma | Pembrolizumab/14 days | Faintness and muscle weakness/ Myocarditis (CK:9786 U/L; CK-MB: 154 U/L; LVEF: 29%; TnT:17.6 ng/mL)/ ECG; CT; EMB | Methylprednisolone (1 g/day for 3 days) followed with immunoglobulin 1 g/kg for 2 days) and tacrolimus | The LVEF gradually improved/Dead | Imai et al., 2019 |
| **Case report** | China | 1 | 43/M | Thymoma | Nivolumab/6 months | Chest pain, dyspnea/Myocarditis (TnI: 6.9 ng/ml; NT-Pro BNP: 1738 pg/mL; CK: 43130 U/L; CK-MB: 1270 U/L/CT; Echocardiography; EMB | Immunoglobulin (300 mg/kg for 4 days) combined with methylprednisone (1 mg/day for 3 days) | No improved/Dead | Chen et al., 2018 |
| **Case report** | Japan | 1 | 77/F | Non-small cell lung cancer | Nivolumab/15 days | Fever and myalgia/ Myocarditis (BNP:88.1 pg/mL; CK: 11297 U/L; CK-MB: 204 U/L; TnT: 1478 ng/L)/ECG; CT | Methylprednisolone 1 g for 3 days) and immunoglobulin (0.4 g/kg/day for 5 days) | Cardiac conduction are improved/Dead | Ono et al., 2022 |
| **Case report** | Japan | 1 | 55/F | Thymoma | Nivolumab/15 days | Motility disorder, diplopia, and dysphagia/ Myocarditis (CK: 13603 IU/L; anti-AChR antibody: 29 nmol/L)/CT; MRI | Immunoglobulin and prednisolone (20 mg/day for 5 days) | Respiratory muscle function was impaired/Dead | Saishu et al., 2022 |
| **Case report** | USA | 1 | 67/M | Metastatic Melanoma | Ipilimumab and nivolumab/10 days | Shortness of breath, cough, and dyspnea/ Myocarditis (TnI: 27.9; NT-ProBNP: 7953 pg/mL; CK: 605U/L; LVEF:20%)/Echocardiogram; ECG; EMB | Methylprednisolone (1 g/day) following with ATG (1.5 mg/kg for 6 doses) | Cardiac function are improved/Dead | Jain et al., 2018 |
| **Case report** | Australia | 1 | 64/F | Glioblastoma | Nivolumab/8 days | Diplopia, myalgias and weakness/ Myocarditis (CK: 3538 U/L; TnI: 8375 ng/L)/ECG; EMB | Methylprednisolone (500 mg/day for 3 days) combined with infliximab (5 mg/day), ATG (500 mg/day for 5 days from day 5) | Clinical symptoms improved/Alive | Tay et al., 2017 |
| **Case report** | Israel | 1 | 53/F | Renal cell carcinoma | Ipilimumab and nivolumab/14 days | Dizziness/Myocarditis (hs-cTnT: 2469 ng/l; NT-proBNP: 8840 pg/ml; LVEF: 30%/ECG; Echocardiography; EMB; TTE | Methylprednisolone (1 g for 3 days) following with ATG (330 mg for 5 doses) | hs-cTnT and NT-proBNP were decline gradually/Alive | Itzhaki Ben Zadok et al., 2019 |
| **Case report** | USA | 8 | 70/M  79/M  61/F  69/M  67/F  83/M  70/M  89/M | Metastatic Melanoma  Metastatic  Melanoma  Breast cancer  Urothelial carcinoma  Metastatic Melanoma  Metastatic  Melanoma  Renal cell carcinoma  Non-small cell  lung cancer | ipilimumab and nivolumab/ 11 days  Pembrolizumab/26days  Durvalumab and  Tremelimumab/ 28 days  Pembrolizumab/13days  Ipilimumab and  Nivolumab/14 days  Nivolumab/31 days  Ipilimumab and  Nivolumab/21 days  Pembrolizumab/32 days | Palpitations, double vision, right ptosis, and pre-syncope/Myocarditis (CK: 3549 U/L; TnI: 3.65 ng/ml; Myoglobin:3307.5 ng/ml)/ ECG; TTE; CMR  Blurred vision, diplopia, fatigue/Myocarditis (TnI:16.45 ng/mL; CK:11953 U/L)/ TTE; ECG;  Ptosis/ Myocarditis (CK:8082 U/L; TnI: 1.03 ng/mL)/ CMR; MRI; ECG  Body pain and weakness/ Myocarditis (CK: 372 U/L; TnI: 1.42 ng/mL)/ TTE; CMR; ECG  Diffuse body weakness, dyspnea, and dysphagia/ Myocarditis (NT-proBNP: 1370 pg/mL; CK: 7244 IU/L; TnI: 12.02 ng/mL)/TTE; CMR; ECG  Fatigue, weakness, chest pain and orthopnea/ Myocarditis (LVEF: 55%; CK: 2886 IU/L; TnI: 0.78 ng/mL)/TTE; CMR  Weakness and fatigue/ Myocarditis (CK:11000 U/L; TnI: 36 ng/mL)/TTE; ECG  Disconjugate gaze and blurred vision/ Myocarditis (TnI: 2.34 ng/mL; CK:3843 U/L)/ TTE; ECG; MRI | Methylprednisolone and ATG  Methylprednisolone, ATG and immunoglobulin  Methylprednisolone  Methylprednisolone and ATG  Methylprednisolone and ATG  Methylprednisolone (1 mg/kg)  Methylprednisolone (1 mg/kg) and prednisone  Methylprednisolone (1 mg/kg) | No sustained improvement/Dead  No clinical improvement/Dead  No clinical improvement/Dead  No clinical improvement/Dead  No clinical improvement/Dead  CK and liver function tests all improved/Dead  Respiratory failure/Dead  CK improved/Dead | Arora et al., 2020 |
| **Case report** | Australia | 1 | 65/F | Esophageal adenocarcinoma | Nivolumab/ 2 weeks | Lightheadedness and dyspnea/ Myocarditis (Hs-TnI:5828 ng/L; CK: 842 U/L)/CT; TTE | Methylprednisolone (1 mg/kg for 4 days) following with ATG. | The clinical state improved/Dead | McDowall et al., 2019 |
| **Case report** | USA | 1 | 75/F | Advanced endometrial cancer | Durvalumab and tremelimumab/ 4 weeks | Neck weakness and dyspnea/ Myocarditis (CPK: 5158 U/L: TnI: 5 ng/mL)/CT; ECG; EMB | Methylprednisolone (1 mg/kg for 3 days) following with mycophenolate mofetil (1000 mg) | The presentation improved/Alive | Mahmood et al., 2018a |
| **Case report** | Canada | 1 | 78/F | Metastatic  melanoma | Pembrolizumab/ 2 weeks | Dyspnea and dysphagia/ Myocarditis (Hs-TnT: 3075 ng/L; NT-proBNP: 4246 pg/ml; CK: 2487 U/L)/CMR; TTE; | Methylprednisolone (2 g for 2 days) combined with mycophenolate mofetil (1.5 g/day), plasmapheresis and abatacept | Cardiac biomarker gradually decreased/Alive | Liu et al., 2020 |
| **Case report** | Canada | 1 | 71/F | Metastatic  melanoma | Pembrolizumab | Shortness of breath and ptosis/ Myocarditis (Hs-TnT: 2373 ng/L; CK: 4300 U/L)/ECG; TTE; | Methylprednisolone (1g/day for 3 days) followed by mycophenolate mofetil (2 g/kg/day for 5 days) and Alemtuzumab (30 mg) | Cardiac biomarker gradually decreased/Alive | Esfahani et al., 2019 |
| **Case report** | USA | 1 | 68/M | Lymphoma | PD-1 and CTL4 inhibitors | Fatigue, generalized malaise, and weakness/ Myocarditis (TnI:9.12 ng/mL; CK-MB: 281ng/mL LVEF: 30%)/ECG; Echocardiography | Mycophenolate mofetil | Cardiac function improved/Alive | Reddy et al., 2017 |
| **Case report** | Canada | 1 | 69/M | Prostate cancer | Pembrolizumab/ 1 month | Fatigue and dyspnea/ Myocarditis (TnI:17.7μg/L; CK: 2437 U/L; LVEF: 30%)/ECG; CMR | Methylprednisolone (2 g for 2 days) combined with mycophenolate mofetil (1 g/day), followed by plasmapheresis (for 2 days) | Cardiac biomarker gradually decreased/Dead | Yogasundaram et al., 2021 |
| **Case report** | France | 1 | 66/F | Lung cancer | Nivolumab/7 days | Ptosis, diplopia and painful paresis/ Myocarditis (TnT:1616 ng/L; NT-proBNP: 4172 ng/L)/CT; Echocardiography; CMR | Methylprednisolone (500 mg/day for 3 days) and abatacept (500 mg, 5 doses) | TnT rapidly decreased/Alive | Salem et al., 2019 |
| **Case report** | Switzerland | 1 | 76/F | Pulmonary adenocarcinoma | Nivolumab/ 7 weeks | Dyspnea/ Myocarditis (NT-proBNP: 32447 ng/L; Hs-TnT: 2674 ng/L; LVEF: 15%)/TTE; ECG | Methylprednisolone (5 mg/kg/day) followed with plasmapheresis, tocilizumab | No improvement/Alive | Doms et al., 2020 |
| **Case report** | Switzerland | 1 | 57/M | Small Cell Lung Cancer | Nivolumab and ipilimumab | Muscular weakness and dyspnea/ Myocarditis (TnT: 1291 ng/L; CK:3778 U/L)/CT; ECG | Methylprednisolone (1 g/day for 1 day and 200 mg/day for 5 days) and tocilizumab (8 mg/kg for 2 doses) | TnT and CK rapidly decreased/Alive | Frigeri et al., 2018 |

ATG: Anti-thymocyte globulin; CK: Creatine kinase; CK-MB: Creatine kinase-myocardial band; CMR: Cardiac magnetic resonance; CPK: Creatine phosphokinase; CPK-MB: Creatinine phosphokinase-myocardial band; CT: Computed tomography; cTnT: Cardiac troponin T; ECG: Electrocardiogram; EMB: Endomyocardial biopsy; MRI: Magnetic resonance imaging; Hs-TnI: High sensitive Troponin I; Hs-TnT: High sensitive Troponin T; NT-proBNP: N-terminal pro-brain natriuretic peptide; PET-MRI: Position emission tomography magnetic resonance imaging; TnI: troponin I; TnT: troponin T; TTE: Transthoracic echocardiography
